# Whole‐exome sequencing in primary pulmonary synovial sarcoma misdiagnosed as mesothelioma: A case report and literature review

**DOI:** 10.1111/1759-7714.14876

**Published:** 2023-04-23

**Authors:** Di Wu, Hongbing Zhang, Xin Li, Jinghao Liu, Minghui Liu, Hongyu Liu, Ming Dong, Jun Chen

**Affiliations:** ^1^ Department of Lung Cancer Surgery Tianjin Medical University General Hospital Tianjin People's Republic of China; ^2^ Tianjin Key Laboratory of Lung Cancer Metastasis and Tumor Microenvironment, Tianjin Lung Cancer Institute Tianjin Medical University General Hospital Tianjin People's Republic of China

**Keywords:** anlotinib, diagnosis and treatment, primary pulmonary synovial sarcoma, whole‐exome sequencing

## Abstract

Synovial sarcoma is a highly malignant tumor that accounts for 10% of all soft tissue sarcomas. Primary pulmonary synovial sarcoma (PPSS) is extremely rare, and its prognosis is poor. A diagnosis is usually established after other primary lung malignancies or metastatic extrathoracic sarcomas have been excluded. Therefore, it is often misdiagnosed. In this study, we report the case of a 38‐year‐old woman who was misdiagnosed as having pleural mesothelioma and finally endured surgery to remove the tumor. The tumor showed SYT‐SSX fusion transcripts and was diagnosed as PPSS after combining histopathological and immunohistochemical analyses. Finally, we determined some biomarkers through whole‐exome sequencing (WES) to improve the diagnosis and treatment strategies.

## Graphical abstract

A sketch of diagnosis and treatment in patient with PPSS. Synovial sarcoma is a highly malignant tumor that accounts for 10% of all soft tissue sarcomas. Primary pulmonary synovial sarcoma (PPSS) is extremely rare, and its prognosis is poor. The diagnosis is usually established after other primary lung malignancies or metastatic extrathoracic sarcomas have been excluded. Therefore, it is often misdiagnosed. In this study, we report the case of a 38‐year‐old woman who was misdiagnosed with pleural mesothelioma and finally endured surgery to remove the tumor. The tumor showed SYT‐SSX fusion transcripts and was diagnosed as PPSS by combining histopathological and immunohistochemical analyses. The patient was treated with anlotinib after surgery and had a good response, but subsequently, drug resistance developed, and the disease progressed. Finally, we determined some biomarkers through whole‐exome sequencing (WES) to improve the diagnosis and treatment strategies.

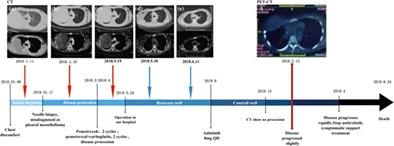

## INTRODUCTION

Synovial sarcoma (SS) originates from primitive multifunctional mesenchymal cells with synovial‐like structures on light microscopy.^1^ It is a soft‐tissue tumor that mainly occurs in the large joints of the extremities and can often lead to lung metastases.^2^ Synovial sarcoma usually have unique genomic characteristics and are driven by a pathognomonic t(X;18) chromesomal translocation and subsequent formation of the SS18;SSX fusion oncogenes. Primary pulmonary synovial sarcoma (PPSS) is extremely rare in lung cancer, accounting for less than 0.5% in patients.^3^, ^4^ Its pathological types mainly include poorly differentiated, biphasic, monophasic epithelia and monophasic fibers (fusiform), of which monophasic is the most common type.^5^ When the tumor is small, a patient generally has no corresponding clinical symptoms. If the patient has symptoms such as chest pain, dyspnea, cough, and pleural effusion, it means the tumor is relatively large, and concurrently, the related imaging features are similar to other intrathoracic malignant tumors.^3^, ^6^ The main metastases are intrathoracic and distant metastases caused by the recurrence of primary lesions. Hematogenous metastasis is the most common, followed by lymph node metastasis, but bone marrow metastasis is rare.^7^ Further, breast^8^ and pancreatic metastases^4^ may occur in a few patients. Diagnosis depends on histological morphology, immunohistochemistry, and chromosome translocation detection.^9^ The prognosis for SS of the lung is poor and the cause of the disease is unclear. The reported 5‐year overall survival (OS) rate is 50%–60%, and the 5‐year disease free survival rate is 40%–60%.^4^, ^10^ Hence, it is necessary to perform multimode treatment through surgery, radiotherapy, and chemotherapy.^5^

Some drugs (ezetimibe, pazopanib, apatinib, etc.) at present are in the clinical trial stages, which provide patients with more treatment options.^11^ Notably, anlotinib, is an oral small molecule receptor tyrosine kinase (RTK) inhibitor, targeting fibroblast growth factor receptors (FGFR1, FGFR2 and FGFR3) and VEGFR1, VEGFR2/KDR, VEGFR3, PDGFR‐α, c‐Kit and Ret. In addition, it can also inhibit tumor cell proliferation, tumor angiogenesis and migration.^12^, ^13^ In the real world, first‐and second‐line treatment of anlotinib is well tolerated and effective in patients with extensive small cell lung cancer.^14^ Anlotinib monotherapy, as a third‐line treatment for patients with advanced non‐small cell lung cancer, has been approved by the United States National Drug Administration.^15^

Until now, most reports in the literature have been short cases that have focused on clinical and imaging features. In our report, we describe a case of PPSS with an emphasis on its clinicopathology and explore the molecular cytogenetic features by whole‐exome sequencing to discover new therapeutics. Concurrently, we systematically review pulmonary synovial sarcoma.

## CASE REPORT

**FIGURE 1:**
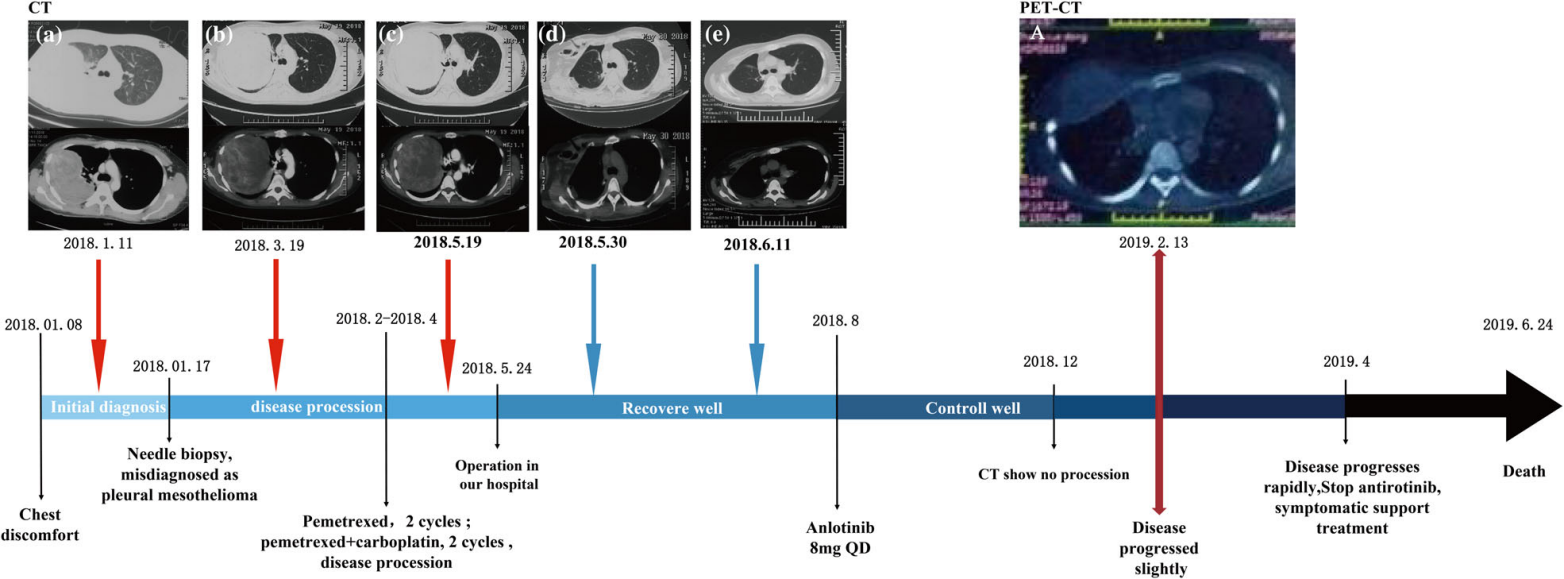
A sketch of the patient's diagnosis and treatment. (a) Computed tomography (CT) showing a transmural mass in the trachea (arrow). (b) Further enlargement of the tumor during chemotherapy (arrow). (c) Preoperative examination of the tumor (arrow). (d) One week after tracheal segmental resection and anastomosis. (e) Six months after surgery. (f) Positron‐emission CT (PET‐CT) showed recurrence of soft tissue tumors around the bronchial stump of the upper lobe and middle lobe of the right lung, and metastasis of the right chest wall and right pleura: thoracic vertebrae.

**FIGURE 2:**
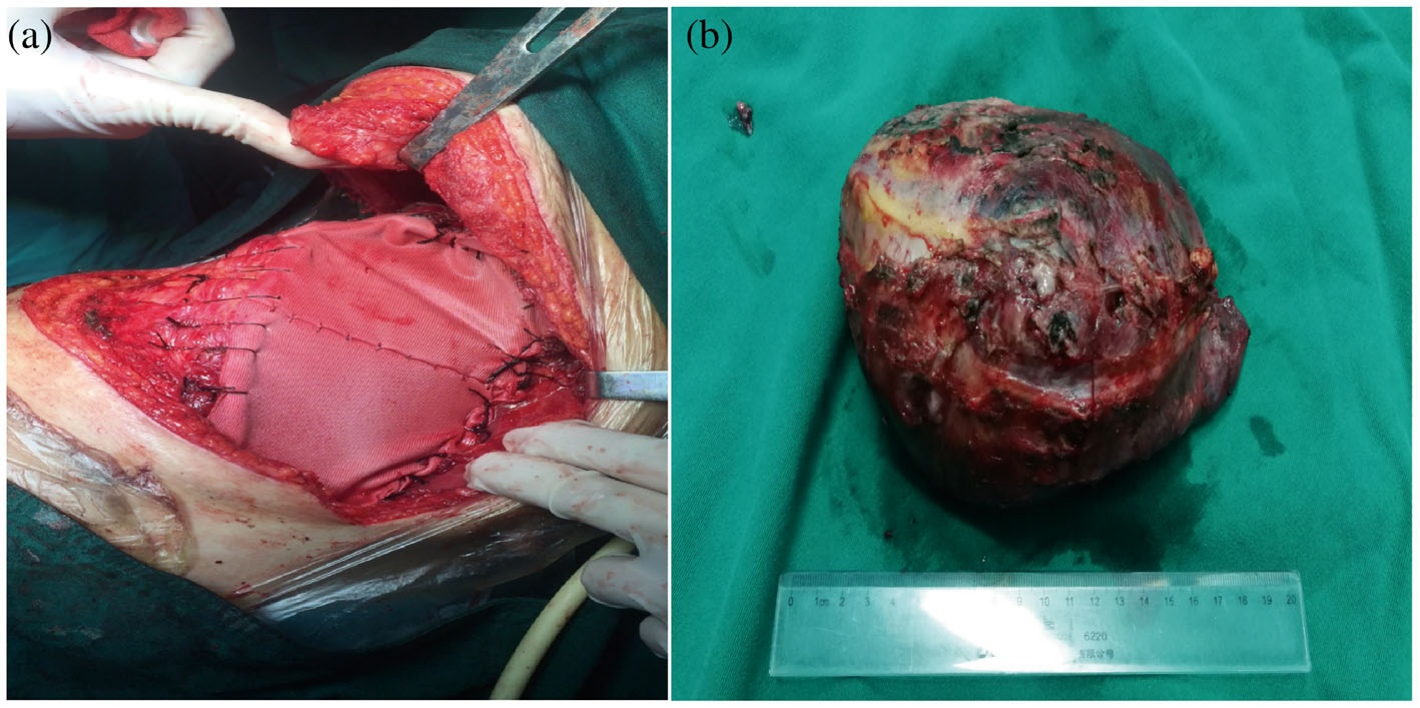
Surgical procedure and specimen. (a) The operation of right upper and middle lobectomy, wedge resection of right lower lobe, partial costectomy of third and fourth rib, systematic lymph node dissection, chest wall reconstruction and closed thoracic drainage. (b) Tumor specimens removed by surgery.

**FIGURE 3:**
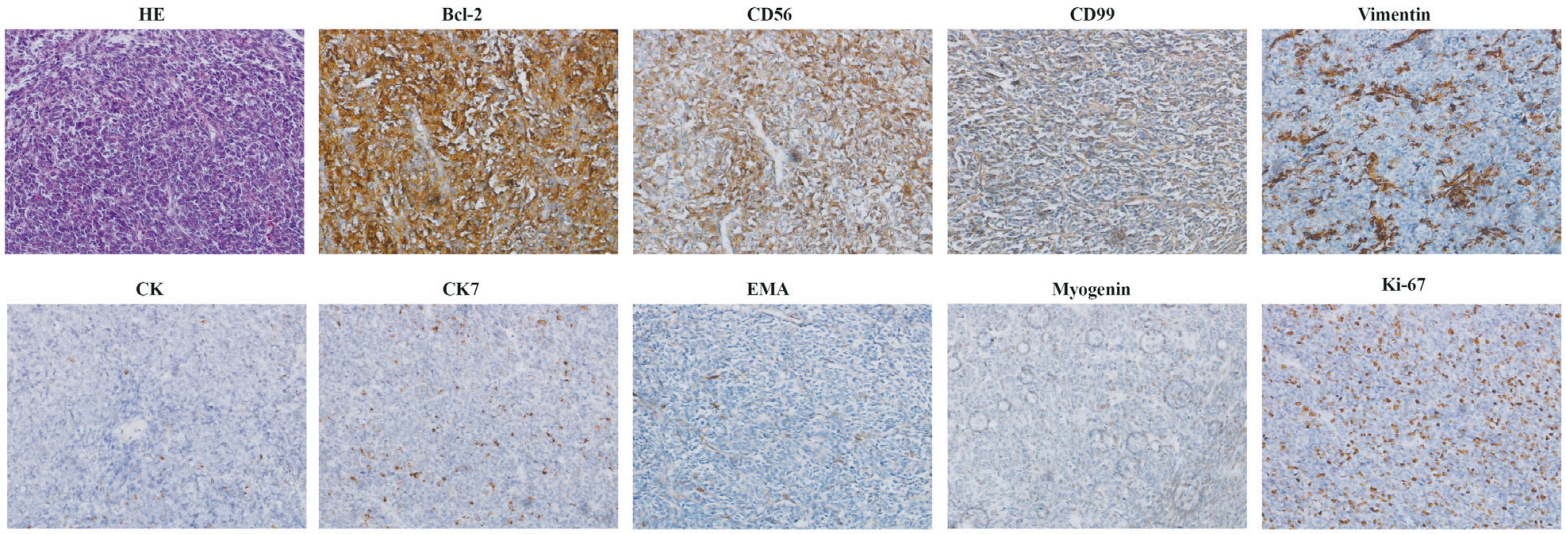
Immunohistochemical staining (magnification, ×200). (a) Hematoxylin and eosin (HE) staining (magnification, ×200) shows that (b) Bc1‐2 and (c) vimentin were positive, (d) CD56 and (e) CD99 were weakly positive, (f) EMA was partially positive, (g) CK and (h) CK7 were scattered positive, (i) myogenin was weakly positive, (j) Ki‐67 index is about 40%.

**FIGURE 4:**
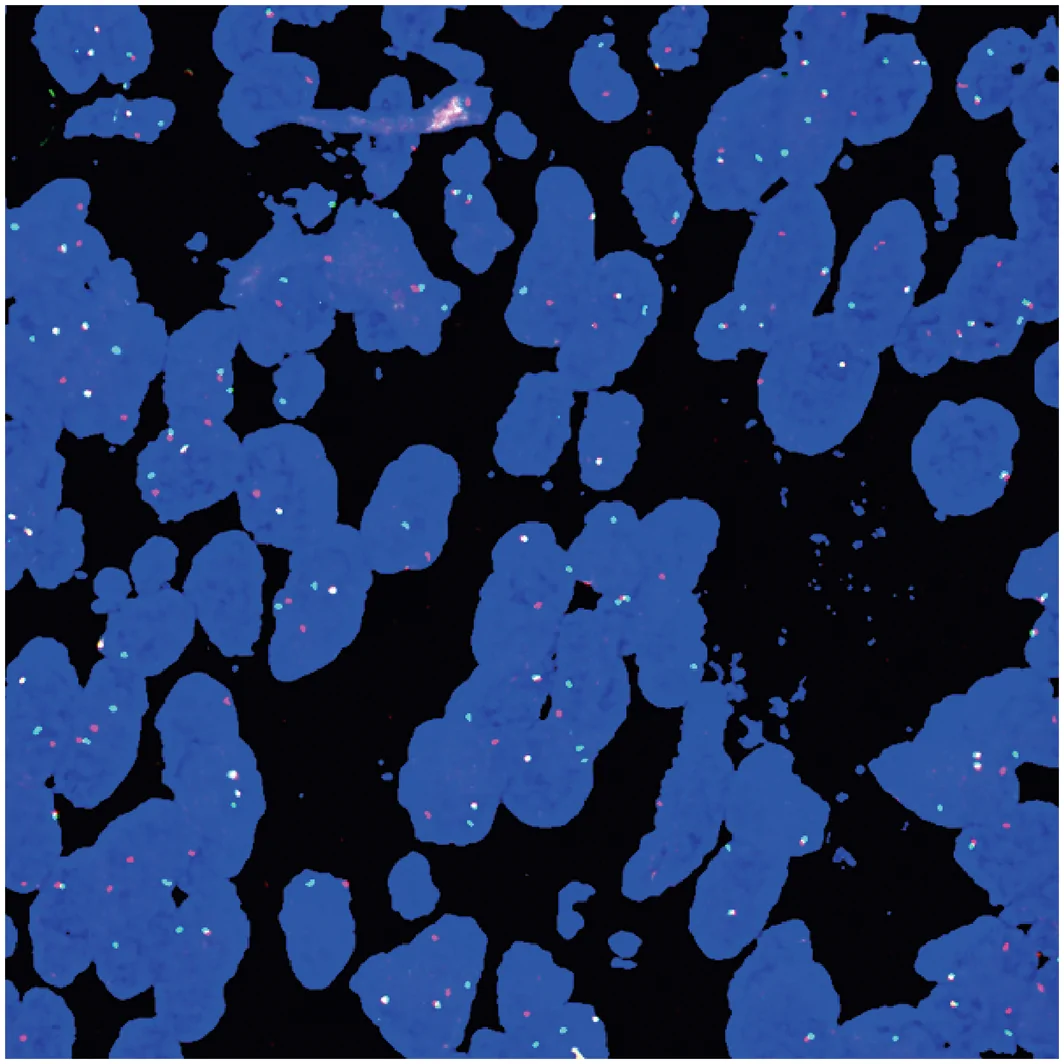
Fluorescence in situ hybridization (FISH) in primary pulmonary synovial sarcoma (PPSS). FISH showed that the SYT split signal was positive in 90% of the tumor cells.

**FIGURE 5:**
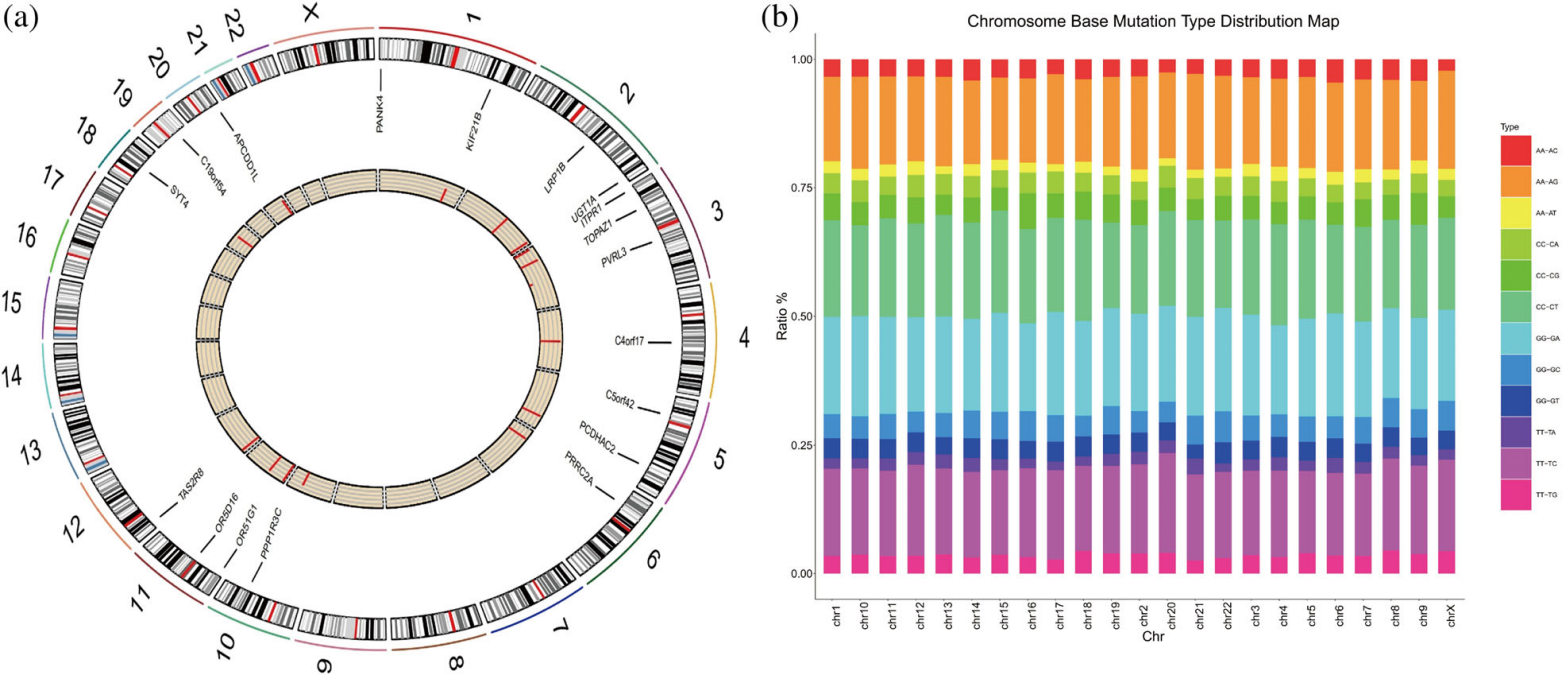
Whole‐exome sequencing (WES) in PPSS. (a) The topic map is 23 chromosome circles, and inner circle is a mutation site tag with a content of more than 3%. Inner circle 4 is a mutation content bar chart. (b) Chromosome base mutation type distribution map: using our analysis of all the mutation sites detected by white blood cells, plus fresh tissue‐specific mutation sites, subchromosome for the number and proportion of each mutation type statistics in the map.

A 38‐year‐old woman was admitted to a local hospital with hemoptysis. The patient had no smoking, asbestos exposure, or malignant family history. She had no other complaints, and her past history was unremarkable, except for spontaneous pneumothorax surgery when she was 19. A review of the systems was noncontributory. Peripheral blood count, baseline serum chemistry screening, and urinalysis were normal on admission, as were tumor biomarker tests (alpha fetoprotein, serum ferritin, carcinoembryonic antigen, antigen 19–9, carbohydrate antigen 24–2, prostate‐specific antigen, neuron‐specific enolase, cytokeratin 19 fragment [CYFRA21‐1], and squamous cell carcinoma antigen) and a purified protein derivative for tuberculosis. The treatment process is shown in Figure 1, and a computed tomography (CT) scan of the chest in the local hospital imaged a 13‐cm pleural‐based mass in the right middle lobe with uneven density, squeezed adjacent pulmonary tissue (Figure 1A). Magnetic resonance imaging (MRI) of the brain, abdominal‐enhanced CT, and bone scintigraphy did not show any metastatic tumors. A core needle biopsy was performed, and the results showed that the histological appearance was biphasic, with a predominant spindle cell pattern, along with necrosis. Immunohistochemical staining showed that CK (cytokeratin), CD34, vimentin, epithelial membrane antibody (EMA), CD5/6 (cytokeratin 5/6), Bcl‐2, Dog‐1, and Ki‐67 (30%+) were positive, while D2‐40, CR, transcription termination factor (TTF‐1), CK7, CD117, CgA, Syn, ER, and PR were negative. Because the patient was misdiagnosed with malignant mesothelioma, the patient received chemotherapy regimen for malignant mesothelioma (Table 1). The patient was initially treated with pemetrexed single‐drug chemotherapy, and the tumor was slightly enlarged after two cycles of re‐examination, after which two cycles of pemetrexed with carboplatin‐combined chemotherapy was administered, and the tumor was not in remission (Figure 1B,C). Hence, the patient was transferred to our hospital for further therapy. Magnetic resonance imaging of the brain, abdominal‐enhanced CT, and bone scintigraphy re‐examination did not show any other abnormalities. However, the chest‐enhanced CT showed that the tumor was slightly larger than the original image. Surgery was undertaken using a right thoracotomy. Right upper and middle lobectomy, wedge resection of the right lower lobe, partial costectomy of the third and fourth ribs, systematic lymph node dissection, chest wall reconstruction, and closed thoracic drainage were performed (Figure 2). No tumor invasion of the bronchial or vascular surgical margins was reported. No postoperative complications were observed (Figure 1D), and the patient was discharged with a close follow‐up. Immunohistochemical staining showed that Bc1‐2 and vimentin were positive; CD56 and CD99 were weakly positive, EMA was partially positive, CK and CK7 were scattered positive, myogenin was scattered weakly positive, P63, P40, TTF‐1, napsin A, Syn, CgA, CD20, CD3, CD79a, desmin, MyoD1, CD138, CD38, S‐100, CD34, HMB45, SOX‐2, and SMA were negative; and Ki‐67 index was about 40% (Figure 3). In addition, fluorescence in situ hybridization (FISH) showed that the SYT split signal was positive in 90% of the tumor cells (Figure 4), indicating the existence of a chromosome translocation of the SYT gene. We conducted exome sequencing of this patient's tumor and explored the molecular structure of the tumor (Figure 5). Postoperative genetic tests confirmed low sensitivity of previous preoperative chemotherapeutic drugs (Table 2).

**TABLE 1:** Comparison of immunostaining of malignant mesothelioma (MM) with synovial sarcoma of the pleura (SS).

Categories	CK	CD34	Vim	EMA	CD5/6	Ki67	Bcl‐2	BerEp4	CEA	B72.3	S100	D2‐40	TTF‐1	CK7	Cga	Syn	AUA1	Des	CD99
MM	+	−	+	+			−	±	±	±	−						−	±	
SS	+	−	+	+			+	±	±	−	±			−	−		±	−	+
X1	F+	V+	+	+	+	30%	+					−	−	−	−	−			
X2	+	−	+	F+		40%	+				−	−	−	+	−	−		−	

*Note*: Pathology and simple identification of our hospital and other hospitals.

Abbreviations: MM, malignant mesothelioma; SS, synovial sarcoma; X1, our patient in other hospital; X2, our patient in our hospital; −, typically absent; +, typically positive; ±, variably positive; F+, focal positive; V+, vascular positive.

The patient refused chemotherapy or radiotherapy after surgery. Considering all aspects of the patient's health, she was given anlotinib (8 mg, QD) three months after surgery. During this period, the patient recovered well without any other discomfort (Figure 1E). The only complaint was regarding the side effects of the drugs, such as hypertension and constipation. The progression‐free survival (PFS) time was about nine months after surgery. The patient underwent a routine postoperative review in February 2019, and PET‐CT indicated multiple metastases (Figure 1F). The soft tissue around the bronchial stump of the upper and middle lobes of the right lung were thickened with increased glucose metabolism. Mass and nodular soft‐tissue density shadow of the right chest wall showed increased glucose metabolism. Local low density shadow of the right latissimus dorsi muscle also showed increased glucose metabolism, which is considered metastasis. At this time, the patient showed no symptoms and continued the current treatment. In April, the patient was hospitalized with chest tightness and shortness of breath, and underwent a comprehensive examination. CT suggested postoperative tumor recurrence of the lung stump, secondary metastasis to the chest wall, pleura and pericardium, and increased right pleural effusion. Adrenal dynamic MRI excluded metastasis, head MRI excluded brain metastases, and lumbar MRI did not exclude lumbar bone metastasis. However, after the ECT examination, bone metastasis was excluded by expert consultation. These results suggest that anlotinib has certain therapeutic effects on small metastases. At this time, the general condition of the patient was acceptable.

In June 2019, the patient's condition rapidly deteriorated, and symptoms of chest tightness and shortness of breath were significantly aggravated. Considering the recurrence of the primary focus of the lung and the invasion of the pericardial tumor, the patient's pleural effusion and pericardial effusion increased significantly. At this time, the patient was in poor condition and near to the deathbed stage. The patient was given symptomatic support treatment, and she finally died on June 24, 2019.

## DISCUSSION

Primary synovial sarcoma of the lung grows slowly and occurs latently, which usually leads to errors in diagnosis and delay in treatment.^16^ The disease usually occurs in young adults with no history of smoking. Patients with the central type are mainly characterized by obstructive pneumonia (fever, cough, and dyspnea) and hemoptysis. Patients with the peripheral type may have no clinical symptoms at first. If it invades the pericardium or pleura, it can cause chest pain;^17^, ^18^, ^19^ irritating symptoms such as pericardium, pleural effusion, and blood/pneumothorax^1^ and rare symptoms including fever, shoulder or back pain, and limb swelling.^20^ Notably, some patients have a history of lung surgery, pleural abrasion, and talc pleurodesis.^21^ However, only a few studies have reported occult lesions after recurrent pneumothorax surgery.^22^ Similarly, this patient had previously undergone surgical treatment for pneumothorax, and PPSS was found 19 years after the operation. Therefore, recurrent pneumothorax and cystic or vesicular lesions need to be further tested for the presence of occult tumor after operation.^22^

**TABLE 2:** The effectiveness of chemotherapy drugs on genes.

Chemotherapeutic drugs	Gene name	Site name	Result	Effectiveness influence	Evidence level
Cisplatin	XRCC1	rs1799782	GG	The effect of drugs is poor.	3
	XRCC1	rs25487	TT	The treatment effect is poor, which is not conducive to prolong the survival time.	2B
Carboplatin	XRCC1	rs25487	TT	The treatment effect is poor, which is not conducive to prolong the survival time.	2B
	XRCC1	rs1799782	GG	The effect of drugs is poor.	3
Oxaliplatin	XRCC1	rs25487	TT	The treatment effect is poor, which is not conducive to prolong the survival time.	2B
	XRCC1	rs1799782	GG	The effect of drugs is poor.	3
Fluorouracil	UMPS	rs2291078	TT	The effect of drug response is good.	4
	UMPS	rs3772810	AA	The effect of drug response is good.	4
	UMPS	rs3772809	AA	The effect of drug response is good.	4
Capecitabine	UMPS	rs2291078	TT	The effect of drug response is good.	4
	UMPS	rs3772809	AA	The effect of drug response is good.	4
	UMPS	rs3772810	AA	The effect of drug response is good.	4

*Note*: The classification of clinical annotations is based on the PharmGKB website https://www.pharmgkb.org/page/clinAnnLevels.

Abbreviations: 1A, confirmed in the clinical genetic pharmacology alliance or genetic pharmacology guidelines, or has been applied in the genetic pharmacology research network and other major health systems; 1B, based on a number of statistics with significant differences, drug annotation studies have confirmed significant associations; 2A, in a number of repetitive studies, higher grades are associated with multidrug combinations, including known important drug genes, which are more likely to have functional meaning; 2B, in multiple repetitive studies, moderate grades are associated with multidrug combinations, but some of these studies are statistically insignificant or have small effects; 3, there is a significant correlation in a single study, or there are many studies, but there is no obvious evidence to show the correlation; 4, the evidence comes from individual research reports, nonsignificant studies or molecular function experimental studies in vitro.

During routine examination, the tumor can show polypoid growth under bronchoscope.^23^ On the imaging findings of CT, PPSS usually shows soft‐tissue mass and pleural mass with round or lobulated boundary, with clear boundary and irregular enhancement.^24^, ^25^ It may also be shown as small nodules and occasionally accompanied by calcification.^26^ Enhanced CT shows the degree of blood triple density of vascular influence tube.^27^ MRI generally shows low‐signal intensity on T1‐weighted images, with or without high‐signal areas indicating bleeding. On T2‐weighted images, swelling and cystic degeneration show uneven signal intensity.^24^ There may be imaging findings of pleural effusion, but mediastinal lymph node enlargement is very rare. PPSS can involve the mediastinum, chest wall, pleura, lung, and pericardium and so on.^28^ Intrathoracic SS has similar manifestations on MRI: blood‐fluid level, triple sign, and grape bowl sign.^28^ In addition, 71% (20/28) showed pleural metastasis, and 48% of the patients with lung metastasis had only ipsilateral lesions.^28^ When the tumor involves the heart, echocardiography and MRI are helpful for preoperative diagnosis.^29^ Therefore, image monitoring provides omni‐directional monitoring for patients and provides an important basis for future treatment plans.^30^

SS grows in four modes: poorly differentiated monophasic subtype, biphasic, monophasic fibrous (spindle cell), and monophasic epithelial.^23^, ^31^ The monophasic type comprises entirely fusiform cells with few cytoplasm, in which a deeply stained nucleus, round, oval, or short fusiform, without obvious division, can be found. A series of morphological features such as interstitial mucinous degeneration, cystic degeneration, ossification, and angiopericytoma are more common in SS.^32^ The biphasic type is formed by a mixed arrangement of fusiform and epithelioid cells. That is, the focal epithelioid region can be seen in the background of fusiform cells, showing fissure‐like, glandular tubular, papillary structure, round nucleus, granular chromatin, occasional nucleolus, light and mucus secretion.^32^ For PPSS, immunohistochemical staining was as follows: CK, CK7, CK16, Bcl‐2, EMA, vimentin, CD56, CD99, and TLE1 were almost positive. The expression of TTF‐1, S‐100, calmodulin (CAM), CD34, desmin, AE1/AE3, EMA, lung cancer markers (TTF‐1 and napsin A), myogenin, neuroendocrine markers (synaptophysin and pheochromogranin A), smooth muscle actin (SMA), signal transduction and transcription activator negative 6 (STAT6), Sox10, calretinin, tumor markers (CD3, CD20, and CD45), synaptophysin, pheochromogranin, P40, FLI‐1, Melan A, WT‐1, P63, NSE, CDX‐2 and vascular tumor markers (TEMS), PD‐1 (programmed cell death protein 1), PD‐L1(programmed death ligand 1), E‐cadherin, β‐catenin, and p53 was almost negative, and the proliferation index of Ki‐67 in tumor cells exceeded 90%.^5^, ^11^, ^32^, ^33^, ^34^, ^35^, ^36^, ^37^, ^38^ Its cytogenetic marker is T (X; 18) (p11; Q11) chromosomal translocation.^23^ The SYT‐SSX fusion gene can be detected by reverse transcriptase polymerase chain reaction (RT‐PCR) or fluorescence in situ hybridization (FISH).^2^, ^11^ In an analysis of 50 people with SS, it was found that the sensitivity, specificity, and negative predictive values of FISH for SS were 82%, 100%, and 75%, respectively.^25^ A study of 243 patients with SS showed that, regardless of tissue type, the prognosis of SS with SYT‐SSX1 fusion transcripts was worse than that of SYT‐SSX2 fusion transcriptions. The 5‐year PFS rates were 42% and 89%, respectively.^39^

In this study, we attempted to explore the potential therapy target using whole‐exome sequencing. In the whole‐exome sequencing data analysis, the mutational status of eight genes significantly differed between the tumor and normal lung tissues among the currently reported valuable genes. In tumor tissues, seven gene amplification mutations included CDK4 (Mutation abundance, MA:1.57), AKT2 (MA:1.65), CCND2 (MA:1.63), MDM2 (MA:1.57), NTRK1 (MA:2.38), AXL (MA:1.65), and KRAS (MA:1.63), as well as the AFF3 gene missense (c.1781_1787del insC). The tumor mutational burden was low at 1.6 muts/Mb. Amplification of the CDK4 and MDM2 genes was found in Ewing's sarcoma.^40^ Recent studies have shown CDK4/6 inhibitors such as palbociclib, which can make sarcoma cells sensitive to Wee1 kinase inhibition through reversible cell cycle arrest.^41^ They suggested that palbociclib may be a potential therapeutic agent for SS. Most studies about KRAS are associated with lung cancer^42^ and colorectal cancer.^43^ In addition, small molecular agents targeting KRAS, such as trametinib and selumetinib for non‐small cell lung cancer, are currently being tested in clinical trials. Their effects on sarcoma remain unknown. AKT2, CCND2, NTRK1, and AXL are associated with multiple tumors and are potential therapeutic targets for SS. However, several groups of gene mutations have been found in this case (the mutation abundance was over 3%), such as LRP1B, TAS2R8, PRRC2A, OR51G1, PCDHAC2, C4orf17, UGT1A, OR5D16, TOPAZ1, SYT4, APCDD1L, ITPR1, PPP1R3C, KIF21B, PVRL3, PANK4, C5orf42, and C19orf54, which may be of certain value to the diagnosis and treatment of this kind of tumor in the future (Table 3).

**TABLE 3:** Whole exon sequencing somatic variation test results (mutation abundance ≥3%).

Gene name	Chromosomes	Base mutation	Mutation abundance
LRP1B	chr2	c.C2812T	52.9
TAS2R8	chr12	c.A320G	51.6
PRRC2A	chr6	c.C3665G	49.6
OR51G1	chr11	c.C814A	49.5
PCDHAC2	chr5	c.1246_1247ins	49.2
C4orf17	chr4	c.G695T	48.7
UGT1A	chr2	c.9358delT	48.5
OR5D16	chr11	c.C876G	46.4
TOPAZ1	chr3	c.T421A	46.4
SYT4	chr18	c.G1126A	45.5
APCDD1L	chr20	c.G1255C	42.9
ITPR1	chr3	c.G7285T	42.2
PPP1R3C	chr10	c.G38A	37.5
KIF21B	chr1	c.2779del	37.2
PVRL3	chr3	c.1123_1124ins	11.8
PANK4	chr1	c.G1735A	4.2
C5orf42	chr5	c.C7477T	3.8
C19orf54	chr19	c.G266A	3.7

*Note*: The mutation sites and abundances of bases with content greater than 3% by whole exon sequencing.

The differential diagnosis of monophasic epithelial SS includes carcinosarcoma, fibrosarcoma, hemangiopericytoma, leiomyosarcoma, spindle cell variant of squamous cell carcinoma, epithelioid schwannoma, malignant melanoma, pleuropulmonary blastoma, malignant mesothelioma, epithelioid sarcoma, and metastatic carcinoma.^5^, ^32^, ^44^, ^45^ Table 4 lists the relatively confusing differential diagnoses.^46^

**TABLE 4:** Differential diagnosis of primary pulmonary synovial sarcoma (PPSS).

Typical morphologic, immunophenotypic, and molecular features associated with the solid pleural tumors																					
Types	Morphologic features	Immunophenotypic features^a^																			Molecular marker
		CK	Calretinin	CK5/6	WT‐1	SMA	Desmin	S100	CD34	CD31	ERG	FLI‐1	CD99	BCL‐2	STAT6	β‐Catenin	TLE1	CAMTA‐1	Vimentin	EMA	
SFT	Bland ovoid cells, patternless architecture, perivascular hyalinization, staghorn vessels, myxoid areas	−	−	−	−	±	−	±	+	−	−	−	+	+	+	−	±	−	+		NAB2‐STAT6 Fusion
PPB	Cystic and/or solid types, immature blastema, spindle cell may be present	−	−	−	−	−	±	±	−	−	−	−	−	−	−	−	−	−	+	+	DICER‐1 mutation
SS	Localized, variable cellularity, overlapping uniform spindle cells, focal calcific deposits	F+	F+	F+	−	−	−	+	−	−	−	−	+	+	−	−	+		+	+	t(X;18) SYT‐SSX fusion
MM	Variable morphology, epithelioid –spindle cell forms	+	+	+	+	±	±	−	−	−	−	−	−	±	−	−	±	−	+	+	NA
X1	NA	F+		F+					V+					+					+	+	NA
X2	NA	+						−	−					+					+	+	t(X;18) SYT‐SSX fusion

Abbreviations: MM, malignant mesothelioma; PPB, pleuropulmonary blastoma; SFT, solitary fibrous tumor; SS, synovial sarcoma; −, typically absent; +, typically positive; ±, variably positive; F+, focal positive; V+, vascular positive.

^a^ It is recognized that in exceptional cases the typical immunophenotype as detailed in the table may be supplemented by the expression of other markers discussed in the text.

A meta‐analysis of 40 patients with mediastinal SS described a median survival of 36 months, while in a large clinicopathological series of 60 patients with intrathoracic SS, 48% of the patients died at the end of the follow‐ups. The average survival time was 23 months; the survival range was 1–78 months; and the median survival time was 35 months.^28^ The prognosis of PPSS is no different from that of other sarcomas, and the overall 5‐year survival rate is 50%;^23^ the long‐term prognosis is poor.^47^ Patients with advanced SS have a survival time of 18–19.7 months;^11^ the overall survival time (OS) of patients with advanced PPSS is generally less than one year because they are inoperable or have metastases.^48^ Risk factors for poor prognosis include male, age over 20 years, incomplete resection, neurovascular invasion, extensive tumor necrosis, tumor maximum diameter over 5 cm, pathological mitosis (>9/10HPF), and SYT‐SSX1 variants.^20^, ^23^, ^25^ Surgical resection is still the first choice, and a negative surgical margin is imperative to prevent local recurrence.^23^, ^25^ The secondary choices are chemotherapy and/or radiotherapy.^25^ Initial standard chemotherapy for advanced or metastatic SS in patients with incomplete resection, lymph node involvement, high‐grade morphology, and large tumor size includes a single anthracycline (mainly doxorubicin) or a combination based on anthracycline, such as dacarbazine and ifosfamide. Regimens containing adriamycin, cyclophosphamide, cisplatin, vincristine, dacarbazine, and other drugs have been shown to be effective in the preoperative and/or postoperative treatment of SS.^7^, ^23^ However, chemotherapy for frail patient may have serious complications and even aggravate the disease progression of patients.^22^ In general, patients may benefit from postoperative adjuvant or neoadjuvant chemotherapy, although the evidence so far is largely inconclusive.^49^ Radiofrequency ablation can be used as an alternative therapy for nonoperative patients.^31^ Through sequential multimode therapy, including surgery, radiosurgery, eribulin, and pazopanib, the survival time of patients can be relatively prolonged.^11^ Prazopanib is a potent and selective multitarget receptor tyrosine kinase inhibitor that blocks tumor growth and inhibits angiogenesis. It has been approved in Japan for treating soft‐tissue sarcoma and kidney cancer.^50^ However, prazopanib can cause tumor necrosis and induce pneumonia or tension pneumothorax, exacerbating disease progression.^11^, ^51^ Until now, it has been reported that apatinib is effective for treating primary synovial sarcoma of the lung^48^ and patients with advanced sarcoma.^52^ Anlotinib, a receptor tyrosine kinase inhibitor, has both antitumor and antiangiogenic activities for advanced lung cancer, and combined with immunotherapy, its curative effect will be better.^13^ A phase IIB randomized trial confirmed that anlotinib can increase the median PFS in patients with advanced soft tissue sarcoma.^15^ Jiang et al. reported a rare case of synovial sarcoma on the right upper leg, and the patient was treated with anlotinib after being diagnosed with lung, cardiac, and adrenal metastases.^53^ For PPSS, anlotinib may inhibit the activation of tumor bypass signaling pathways and delay tumor growth through, but its subsequent drug resistance is also the main problem. Therefore, based on previous clinical trials, immunotherapy can be used to delay the progression of the disease in the later stage of treatment. This requires further exploration in the later stages.

In conclusion, for a previous medical history, young patients with pneumothorax after surgical treatment may have the possibility of occult pulmonary synovial sarcoma, so we need to pay more attention to it. PPSS is a rare disease, and its diagnosis is relatively complicated. FISH is the gold standard for the diagnosis of synovial sarcomas, especially synovial sarcomas in special parts, which depends very much on the judgment of pathologists. If necessary, omni‐directional immunohistochemistry and gene detection should be used to reduce the misdiagnosis of the disease.^54^, ^55^ The prognosis of the disease is very poor. For early patients, early detection, diagnosis, and comprehensive treatment based on operations should be carried out. For patients with advanced lung cancer, neoadjuvant therapy, chemotherapy, targeted therapy, immunotherapy, and radiofrequency ablation have broad prospects for treating them, and their treatment schemes should be further explored and improved. In this case, the patient also achieved good PFS with anlotinib, so it can also be used as a potential treatment option for advanced patients. Gene mutations such as LRP1B, TAS2R8, PRRC2A, OR51G1, PCDHAC2, C4orf17, UGT1A, OR5D16, TOPAZ1, SYT4, APCDD1L, ITPR1, PPP1R3C, KIF21B, PVRL3, PANK4, C5orf42, and C19orf54 play important roles in the diagnosis and treatment of the disease. They help in expounding the molecular biological laws of the occurrence and development of the disease, they also enrich the possible pathogenic factors of the disease. Overall, the results of the whole‐exome sequencing further reveal that several somatic mutations may cause PPSS, which will play a certain role in promoting drug research and development of the disease in the future.

## AUTHOR CONTRIBUTIONS

Provided medical care for the patients and collected the data: Di Wu, Hongbing Zhang, Xin Li, Jinghao Liu, Minghui Liu, Ming Dong, Jun Chen. Quality control of data: Di Wu, Hongyu Liu, Ming Dong, Jun Chen. Manuscript preparation: Di Wu, Ming Dong and Jun Chen. Manuscript editing and reviewing: all authors. All authors read and approved the final manuscript.

## CONFLICT OF INTEREST STATEMENT

The authors declare that the research was conducted in the absence of any commercial or financial relationships that could be construed as a potential conflict of interest.
